# Total Body Irradiation or Chemotherapy Conditioning in Childhood ALL: A Multinational, Randomized, Noninferiority Phase III Study

**DOI:** 10.1200/JCO.20.02529

**Published:** 2020-12-17

**Authors:** Christina Peters, Jean-Hugues Dalle, Franco Locatelli, Ulrike Poetschger, Petr Sedlacek, Jochen Buechner, Peter J. Shaw, Raquel Staciuk, Marianne Ifversen, Herbert Pichler, Kim Vettenranta, Peter Svec, Olga Aleinikova, Jerry Stein, Tayfun Güngör, Jacek Toporski, Tony H. Truong, Cristina Diaz-de-Heredia, Marc Bierings, Hany Ariffin, Mohammed Essa, Birgit Burkhardt, Kirk Schultz, Roland Meisel, Arjan Lankester, Marc Ansari, Martin Schrappe, Arend von Stackelberg, Adriana Balduzzi, Selim Corbacioglu, Peter Bader

**Affiliations:** ^1^ St. Anna Children's Hospital, Children's Cancer Research Institute, University Vienna, Vienna, Austria; ^2^ Hôpital Robert Debré, GH APHP-Nord Université de Paris, Paris, France; ^3^ Department of Pediatric Hematology and Oncology, IRCCS Ospedale Pediatrico Bambino Gesù, Sapienza University of Rome, Rome, Italy; ^4^ Children's Cancer Research Institute, Vienna, Austria; ^5^ Department of Pediatric Hematology and Oncology, Motol University Hospital, Prague, Czech Republic; ^6^ Department of Pediatric Hematology and Oncology, Oslo University Hospital, Oslo, Norway; ^7^ The Children`s Hospital at Westmead, Sydney, Australia; ^8^ Hospital de Pediatría, Buenos Aires, Argentina; ^9^ Copenhagen University Hospital Rigshospitalet, Copenhagen, Denmark; ^10^ Children's Hospital, University of Helsinki, Helsinki, Finland; ^11^ National Institute of Children's Diseases, Bratislava, Slovakia; ^12^ Belarusian Research Center for Pediatric Oncology, Hematology and Immunology, Borovlyani, Belarus; ^13^ Schneider Children's Medical Center of Israel, Sackler Faculty of Medicine, Tel Aviv University, Petach-Tikva, Israel; ^14^ Universitäts-Kinderspital, Zurich, Switzerland; ^15^ Skåne University Hospital, Lund, Sweden; ^16^ Alberta Children's Hospital Calgary, Calgary, Alberta, Canada; ^17^ Hospital Universitari Vall d’Hebron, Barcelona, Spain; ^18^ Princess Máxima Center for Pediatric Oncology, Bilthoven, the Netherlands; ^19^ University of Malaya, Kuala Lumpur, Malaysia; ^20^ King Abdullah Specialist Children's Hospital, King Abdullah International Medical Research Center, King Saud Bin Abdulaziz University for Health Sciences, Riyadh, Saudi Arabia; ^21^ Children's University Hospital Münster, Münster, Germany; ^22^ University of British Columbia, Vancouver, British Columbia, Canada; ^23^ Division of Pediatric Stem Cell Therapy, Department of Pediatric Oncology, Hematology and Clinical Immunology, Medical Faculty, Heinrich-Heine-University, Duesseldorf, Germany; ^24^ Willem-Alexander Children's Hospital, Leiden, the Netherlands; ^25^ Geneva University Hospital, Geneva, Switzerland; ^26^ Universitätsklinikum Schleswig-Holstein, Kiel, Germany; ^27^ Charité University Hospital Berlin, Berlin, Germany; ^28^ Università degli Studi di Milano—Fondazione MBBM, Monza, Italy; ^29^ Universitätsklinikum Regensburg, Regensburg, Germany; ^30^ Goethe University, University Hospital Frankfurt, Department for Children and Adolescents, Division for Stem Cell Transplantation, Immunology and Intensive Care Medicine, Frankfurt am Main, Germany

## Abstract

Total body irradiation (TBI) before allogeneic hematopoietic stem cell transplantation (HSCT) in pediatric patients with acute lymphoblastic leukemia (ALL) is efficacious, but long-term side effects are concerning. We investigated whether preparative combination chemotherapy could replace TBI in such patients.

**PATIENTS AND METHODS:**

FORUM is a randomized, controlled, open-label, international, multicenter, phase III, noninferiority study. Patients ≤ 18 years at diagnosis, 4-21 years at HSCT, in complete remission pre-HSCT, and with an HLA-compatible related or unrelated donor were randomly assigned to myeloablative conditioning with fractionated 12 Gy TBI and etoposide versus fludarabine, thiotepa, and either busulfan or treosulfan. The noninferiority margin was 8%. With 1,000 patients randomly assigned in 5 years, 2-year minimum follow-up, and one-sided alpha of 5%, 80% power was calculated. A futility stopping rule would halt random assignment if chemoconditioning was significantly inferior to TBI (EudraCT: 2012-003032-22; ClinicalTrials.gov: NCT01949129).

**RESULTS:**

Between April 2013 and December 2018, 543 patients were screened, 417 were randomly assigned, 212 received TBI, and 201 received chemoconditioning. The stopping rule was applied on March 31, 2019. The median follow-up was 2.1 years. In the intention-to-treat population, 2-year overall survival (OS) was significantly higher following TBI (0.91; 95% CI, 0.86 to 0.95; *P* < .0001) versus chemoconditioning (0.75; 95% CI, 0.67 to 0.81). Two-year cumulative incidence of relapse and treatment-related mortality were 0.12 (95% CI, 0.08 to 0.17; *P* < .0001) and 0.02 (95% CI, < 0.01 to 0.05; *P* = .0269) following TBI and 0.33 (95% CI, 0.25 to 0.40) and 0.09 (95% CI, 0.05 to 0.14) following chemoconditioning, respectively.

**CONCLUSION:**

Improved OS and lower relapse risk were observed following TBI plus etoposide compared with chemoconditioning. We therefore recommend TBI plus etoposide for patients > 4 years old with high-risk ALL undergoing allogeneic HSCT.

## INTRODUCTION

Total body irradiation (TBI) is widely used in conditioning regimens for patients with acute lymphoblastic leukemia (ALL) undergoing allogeneic hematopoietic stem cell transplantation (HSCT).^1-3^ For children with high-risk ALL, an allogeneic HSCT from an HLA-identical sibling donor (MSD) or HLA-compatible related or unrelated matched donor (MD)^4-7^ or mismatched donor,^8^ conditioned with TBI and etoposide has resulted in excellent overall and leukemia-free survival.

**CONTEXT**

**Key Objective**
Total body irradiation (TBI) before allogeneic hematopoietic stem cell transplantation (HSCT) in pediatric patients with acute lymphoblastic leukemia (ALL) is efficacious but can have long-term side effects. With a view to improving options for patients, the FORUM randomized, controlled, open-label, international, multicenter, phase III trial was designed to investigate whether preparative combination chemotherapy is noninferior to TBI.
**Knowledge Generated**
After random assignment of 417 pediatric patients with high-risk ALL, a futility stopping rule was applied because patients receiving chemoconditioning with fludarabine, thiotepa, and either busulfan or treosulfan had inferior overall survival (OS) to those receiving TBI plus etoposide. Two-year OS was 0.91 (95% CI, 0.86 to 0.95; *P* < .0001) following TBI versus 0.75 (95% CI, 0.67 to 0.81) following chemoconditioning.
**Relevance**
Of relevance to clinical practice, the authors recommend TBI plus etoposide for patients > 4 years old with high-risk ALL undergoing allogeneic HSCT because of the higher survival and lower relapse risk observed in comparison with chemoconditioning.

A small, randomized, controlled trial found significantly higher event-free survival (EFS) with TBI, etoposide, and cyclophosphamide versus busulfan, etoposide, and cyclophosphamide conditioning in pediatric ALL patients receiving an unrelated donor HSCT, but a nonsignificant difference for those receiving a related donor HSCT.^9^ Moreover, a meta-analysis in patients with leukemia found significantly lower treatment-related mortality (TRM) with TBI-based versus busulfan-based conditioning.^10^ However, TBI has lifelong adverse effects. Impairment of growth, gonadal function, and cognitive function, cataracts, and secondary malignancies are more frequent after TBI than irradiation-free conditioning regimens.^11-13^

New chemotherapeutic agents and combinations have brought promise of fewer acute and late effects compared with TBI that may outweigh the risk of leukemic reappearance; in particular, fludarabine, thiotepa, targeted busulfan, and treosulfan show promise.^14-17^ Head-to-head prospective comparisons of chemoconditioning versus TBI-containing regimens have not evaluated disease-free survival or acute and long-term adverse events (AEs).

We conducted this prospective, randomized, controlled trial to investigate whether optimal chemoconditioning regimens^14,15,18,19^ could replace TBI in pediatric patients with high-risk ALL.

## PATIENTS AND METHODS

### Study Design

The For Omitting Radiation Under Majority age (FORUM) study is an international, randomized, open-label, phase III study (EudraCT 2012-003032-22; ClinicalTrials.gov: NCT01949129). We report on the randomly assigned part, conducted in 88 centers in 21 countries, with the primary aim to test noninferiority of chemoconditioning versus TBI with regard to overall survival (OS).

The study was designed by experts from ALL-Frontline and Relapse study groups and members of several pediatric transplantation groups. The Protocol (online only) and statistical analysis plan were approved by the investigational review board or independent ethics committee and national authorities for each center. The trial was performed in accordance with the Declaration of Helsinki principles.

An Independent Data Monitoring Committee (IDMC) periodically reviewed safety and efficacy data. Per stopping rules, random assignment would end if chemoconditioning was significantly worse than control (5% level; log-rank test). The IDMC recommended random assignment suspension in December 2018. After extensive data analyses, we confirmed the findings and stopped random assignment on March 31, 2019.

### Patients

Patients eligible for random assignment had high-risk ALL, were ≤ 18 years old at initial diagnosis, 4-21 years old at HSCT, had an indication for allogeneic HSCT, were in complete morphological remission pre-HSCT, and had an MSD or MD allelic matched at nine or 10 out of 10 HLA loci. Exclusion criteria included prior HSCT, pre-HSCT cranial radiation > 18 Gy at any time or > 12 Gy in the previous 24 months, pre-existing severe organ toxicities, pregnancy, or secondary malignancy. Written informed consent was provided by patients, parents, or legal guardians.

The recommended stem cell source was bone marrow or cord blood from an MSD, or bone marrow, peripheral blood stem cells, or cord blood from an MD.

### Procedures

Patients were randomly assigned 1:1 either to TBI plus 60 mg/kg intravenous etoposide (1.8 g/m^2^; upper total dose 3.6 g) once on day 3 before HSCT or to intravenous fludarabine 30 mg/m^2^ once a day over 5 days, thiotepa 5 mg/kg twice a day for 1 day, and either treosulfan 14 g/m^2^ once a day for 3 days or busulfan over 4 days. National coordinators chose busulfan or treosulfan. Busulfan was dosed once, twice, or four times a day according to local guidelines, age, and body weight, commonly with therapeutic drug monitoring and pharmacokinetic dose adjustment. TBI was delivered from a linear accelerator at 12 Gy in six fractions over 3 days with lung shielding at 10 Gy.

Graft-versus-host disease (GVHD) prophylaxis was contingent upon donor type and stem cell source. MSD recipients received cyclosporine A only, while MD recipients also received methotrexate and antithymocyte globulin.

Complete remission (CR) was defined as ≤ 5% bone marrow blasts and no evidence of extramedullary disease. Minimal residual disease (MRD) in bone marrow was assessed by flow cytometry or polymerase chain reaction (PCR) in the 2 weeks before conditioning therapy. MRD positivity was defined as > 10^−3^ for flow cytometry or > 10^−4^ for PCR. Relapse was defined as ≥ 5% leukemic blasts in bone marrow and/or detection in extramedullary sites (eg, cerebrospinal fluid, testes, and ovary).

Clinicians assessed patients for AEs, adverse reactions, serious AEs, and suspected expected or unexpected serious adverse reactions per good clinical practice guidelines. AEs were graded by the National Cancer Institute Common Terminology Criteria for Adverse Events, version 3.0. Serious AEs were fatal, life-threatening, or other medically important serious events, leading to intensive care unit admission (ie, grade 3-4). Acute and chronic GVHD (aGVHD and cGVHD, respectively) were assessed at each visit and graded as previously described.^20^

### Outcomes

The primary end point was OS from random assignment date. Death from any cause was considered an event. Patients lost to follow-up without an event were censored at last follow-up. Secondary end points included EFS, cumulative incidence of relapse (CIR), TRM, aGVHD, cGVHD, toxicity at day 100, and GVHD-free, relapse-free survival.

### Statistical Analysis

The design was a noninferiority study with an 8% margin. With a sample size of 1,000 patients randomly assigned in 5 years, 2-year minimum follow-up, and a one-sided alpha of 5%, 80% power was calculated. Early stopping made follow-up too short for the intended primary analysis to be feasible.

**FIG 1.**
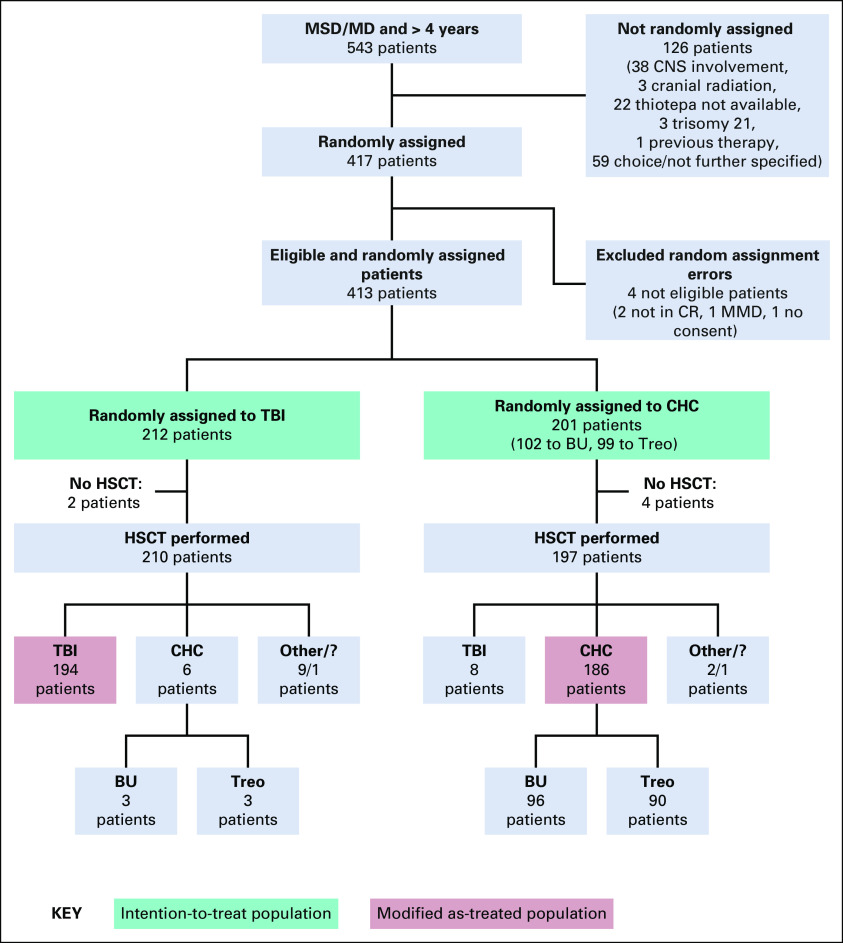
Patient enrollment, random assignment, and adherence to protocol. Other: Variable modifications of the given conditioning regimen due to medical reasons or center/parental decision. BU, busulfan; CHC, chemo-conditioning; CR, complete remission; MD, human leukocyte antigen (HLA)–compatible related or unrelated matched donor; MMD, mismatched donor; MSD, HLA-identical sibling donor; TBI, total body irradiation; Treo, treosulfan.

In FORUM, patients randomly assigned to TBI yet who received chemoconditioning could be anticipated to be at lower risk than patients randomly assigned to chemoconditioning yet who received TBI, meaning that a per-protocol analysis might favor chemoconditioning. Accordingly, the ITT population was protocol-specified for the primary analysis (see the Data Supplement, online only). The results reported are from ITT analyses unless otherwise indicated (data cutoff November 2019; random assignment errors excluded; Fig 1). A secondary modified as-treated analysis compared outcomes following TBI, treosulfan-based chemoconditioning, and busulfan-based chemoconditioning, excluding some protocol violations (Fig 1). AEs were assessed in the modified as-treated population.

OS, EFS, and GVHD-free, relapse-free survival were estimated with Kaplan-Meier methodology and compared using the logrank test. Two-year estimates and 95% CIs used log transformation (α = 5%). For the modified as-treated and univariate analyses, pairwise comparisons were performed if the global *P* value was significant. For multivariable analyses, Cox regression explored the impact of risk factors and conditioning type on OS and EFS. In the presence of monotone likelihoods, Firth correction was used. Cox regression formally tested stratification factors and interactions. Proportions of patients with grade 3-4 aGVHD and grade 3-4 AEs at day 100 were compared using a χ^2^ test. Cumulative incidences of relapse, TRM, and cGVHD were estimated accounting for competing events and compared using Gray's test. Multivariable evaluation of relapse incidence used the Fine and Gray model. Subgroup analyses were ad hoc. Median follow-up was estimated using the inverse Kaplan-Meier method.

## RESULTS

Between April 18, 2013, and March 31, 2019, a total of 543 patients were screened and 413 were randomly assigned (Fig 1), mainly in Germany and France (Data Supplement). Two hundred and twelve patients were randomly assigned to TBI, and 201 were randomly assigned to chemoconditioning. Compliance with random assignment was 92%. Table 1 shows patient demographics and baseline characteristics, which were well balanced between groups. Sixty-five percent of participants were male, 72% had B-cell precursor ALL, 73% had an MD, and 82% underwent bone marrow transplantation. Fifty-four percent of participants were in first complete remission (CR1) at inclusion. Of patients assessed for MRD by PCR or flow cytometry, 43% were positive and 57% were negative.

**TABLE 1.**
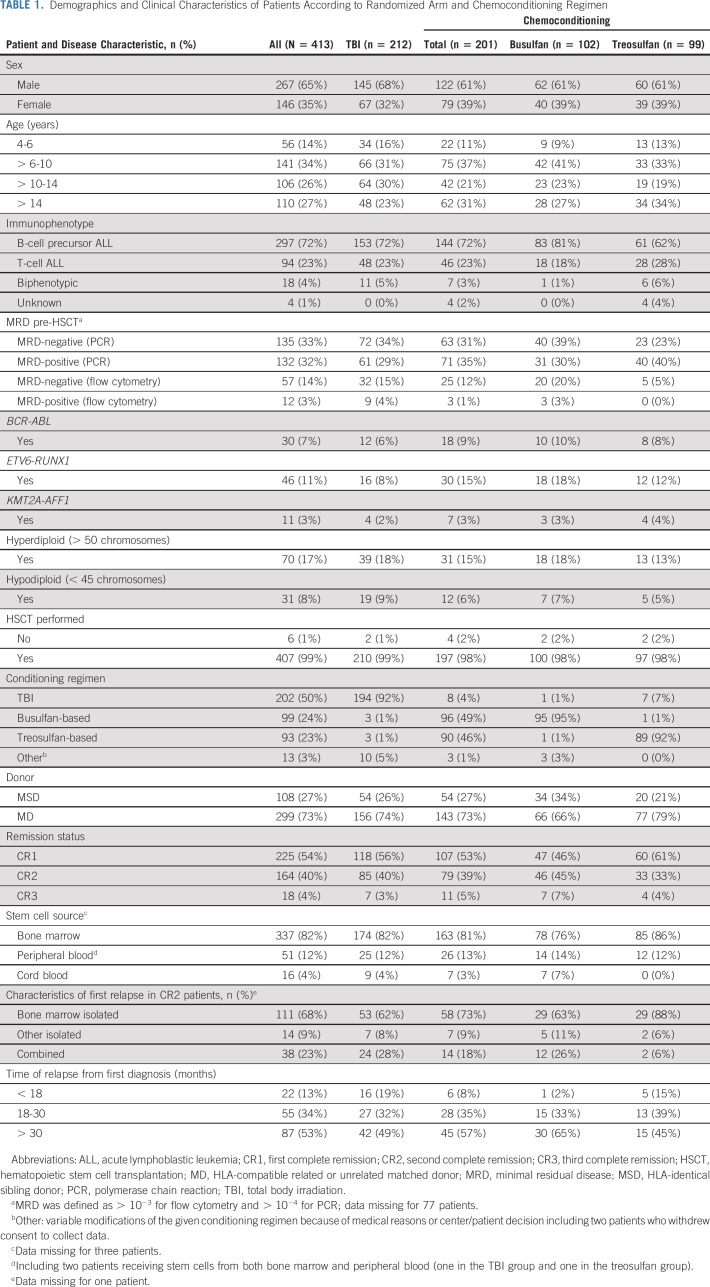
Demographics and Clinical Characteristics of Patients According to Randomized Arm and Chemoconditioning Regimen

**FIG 2.**
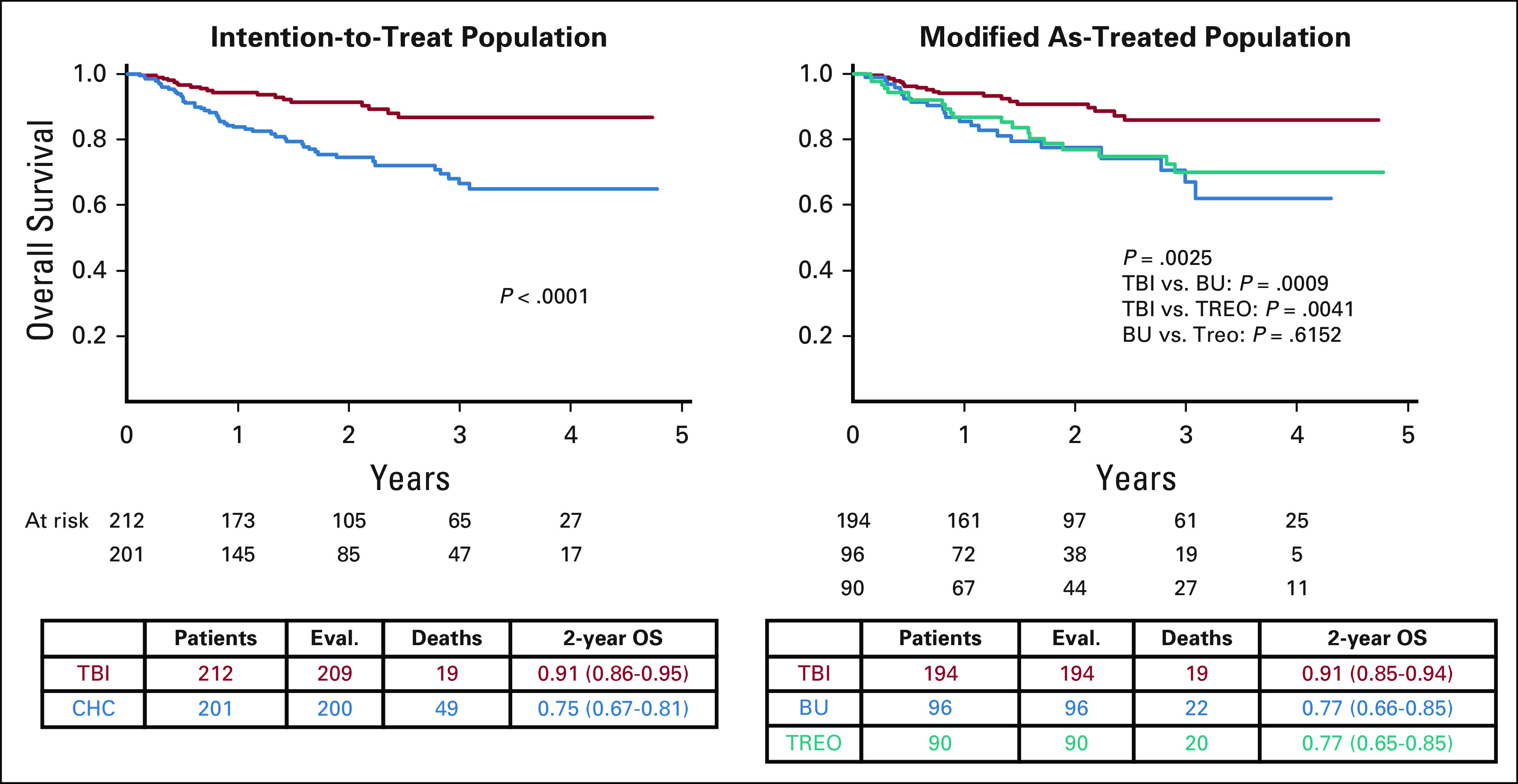
Primary end point: Overall survival. BU, busulfan; CHC, chemo-conditioning; CIR, cumulative incidence of relapse; EFS, event-free survival; OS, overall survival; TBI, total body irradiation; TREO, treosulfan; TRM, treatment-related mortality.

With a median follow-up of 2.1 years (range, 1 month to 6 years), OS was significantly higher following TBI versus chemoconditioning, with a 2-year probability of OS of 0.91 (95% CI, 0.86 to 0.95) versus 0.75 (95% CI, 0.67 to 0.81), respectively (*P* < .0001; Fig 2). Two-year EFS was significantly higher following TBI versus chemoconditioning (0.86 [95% CI, 0.79 to 0.90] *v* 0.58 [95% CI, 0.50 to 0.66], respectively; *P* < .0001; Fig 2).

For 224 patients transplanted in CR1, 2-year OS and EFS were 0.85 (95% CI, 0.79 to 0.90) and 0.80 (95% CI, 0.73 to 0.85), respectively, with both being significantly higher in the TBI versus chemoconditioning arm (Data Supplement). For 87 patients transplanted in second complete remission (CR2) who relapsed > 30 months after diagnosis, 2-year OS and EFS were 0.89 (95% CI, 0.78 to 0.95) and 0.69 (95% CI, 0.56 to 0.79), respectively; both end points were significantly higher in the TBI versus chemoconditioning arm (Data Supplement). In 69 patients with a *BCR-ABL* mutation, *KMT2A-AFF1* translocation, or blast cell hypodiploidy (< 45 chromosomes), 2-year EFS (but not OS) was significantly higher with TBI versus chemoconditioning (0.89 [95% CI, 0.71 to 0.97] *v* 0.60 [95% CI, 0.36 to 0.78], respectively; *P* = .0182).

Two-year TRM was 0.02 (95% CI, < 0.01 to 0.05) following TBI and 0.09 (95% CI, 0.05 to 0.14) following chemoconditioning (*P* = .0269). Of the TBI, busulfan-containing chemoconditioning, and treosulfan-containing chemoconditioning groups, 7/194 (3.6%), 7/96 (7.3%), and 9/90 (10.0%) patients, respectively, died without relapse (modified as-treated analysis; Table 2; Fig 2).

**TABLE 2.**
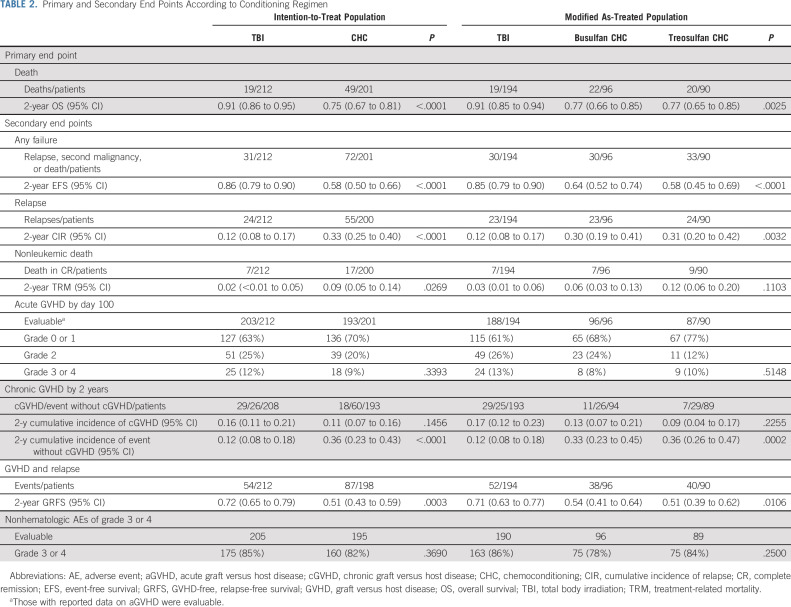
Primary and Secondary End Points According to Conditioning Regimen

The results for OS, EFS, and TRM were superimposable in modified as-treated analyses to those observed in the ITT analyses. No significant differences between the two chemoconditioning regimens were observed (Fig 2).

The most frequent reason for treatment failure was relapse, commonly in bone marrow (56/70 patients). Two-year CIR was 0.12 (95% CI, 0.08 to 0.17) following TBI and 0.33 (95% CI, 0.25 to 0.40) following chemoconditioning (*P* < .0001; Table 2; Fig 2). This difference remained significant when patients were stratified by remission status (Data Supplement).

Of 67 patients who relapsed after HSCT, 38 died (12 after TBI, 15 busulfan, and 11 treosulfan); 39 received salvage therapy with curative intent. In patients who relapsed post-transplant, there was no significant difference in OS between conditioning regimens (modified as-treated analysis; Data Supplement).

There were no suspected unexpected serious adverse reactions. Regimens were associated with substantial degrees of reported toxicity, as expected. The most common grade 3-4 AEs at day 100 in all arms were cytopenia, mucositis, nausea, and infection (Data Supplement).

In the modified as-treated analysis, TRM occurred in 7/194 patients following TBI, 7/96 patients after busulfan-based chemoconditioning, and 9/90 patients after treosulfan-based chemoconditioning. The most common causes were infection, organ toxicity, or GVHD, with no significant difference between conditioning regimens (Data Supplement).

There was no significant difference in the proportion of patients experiencing aGVHD or cGVHD between arms (Table 2). Of 396 patients evaluable for aGVHD, 263 experienced no or grade 1 aGVHD, 90 developed grade 2 aGVHD, and 43 developed grade 3-4 aGVHD by day 100 (Table 2).

Two-year EFS across the whole study population stratified by aGVHD grade 0-1, 2, and 3-4 was 0.72 (95% CI, 0.64 to 0.78), 0.85 (95% CI, 0.73 to 0.92), and 0.54 (95% CI, 0.36 to 0.69), respectively (*P* = .0041). Two-year TRM was significantly higher for patients with grade 3-4 aGVHD versus those with grade 0-1 or 2 (0.22 [95% CI, 0.10 to 0.37], 0.04 [95% CI, 0.02 to 0.08], and 0.02 [95% CI, < 0.01 to 0.07], respectively; *P* < .0001).

No significant difference in the incidence and severity of aGVHD was observed when patients were stratified by donor type or conditioning regimen (Data Supplement). Two-year cumulative incidence of cGVHD did not significantly differ between arms (Table 2).

The probability of GVHD-free, relapse-free survival at 2 years was 0.72 (95% CI, 0.65 to 0.79) following TBI and 0.51 (95% CI, 0.43 to 0.59) following chemoconditioning (*P* < .0003; Table 2; Data Supplement).

In univariate analyses, MSD and CR1 significantly predicted better OS and EFS. Female patients had a lower probability of EFS (n = 145; 0.64 [95% CI, 0.54 to 0.72]) versus male patients (n = 264; 0.77 [95% CI, 0.70 to 0.82]; *P* = .016) (Data Supplement). Analysis of OS by risk factor and conditioning regimen is shown in Figure 4 and the Data Supplement. A significant interaction was observed when age and conditioning therapy were analyzed, with the beneficial effect of TBI on OS strongest in patients of age 6-14 years. No significant interactions between risk factors and conditioning regimens were observed for EFS (Data Supplement).

**FIG 3.**
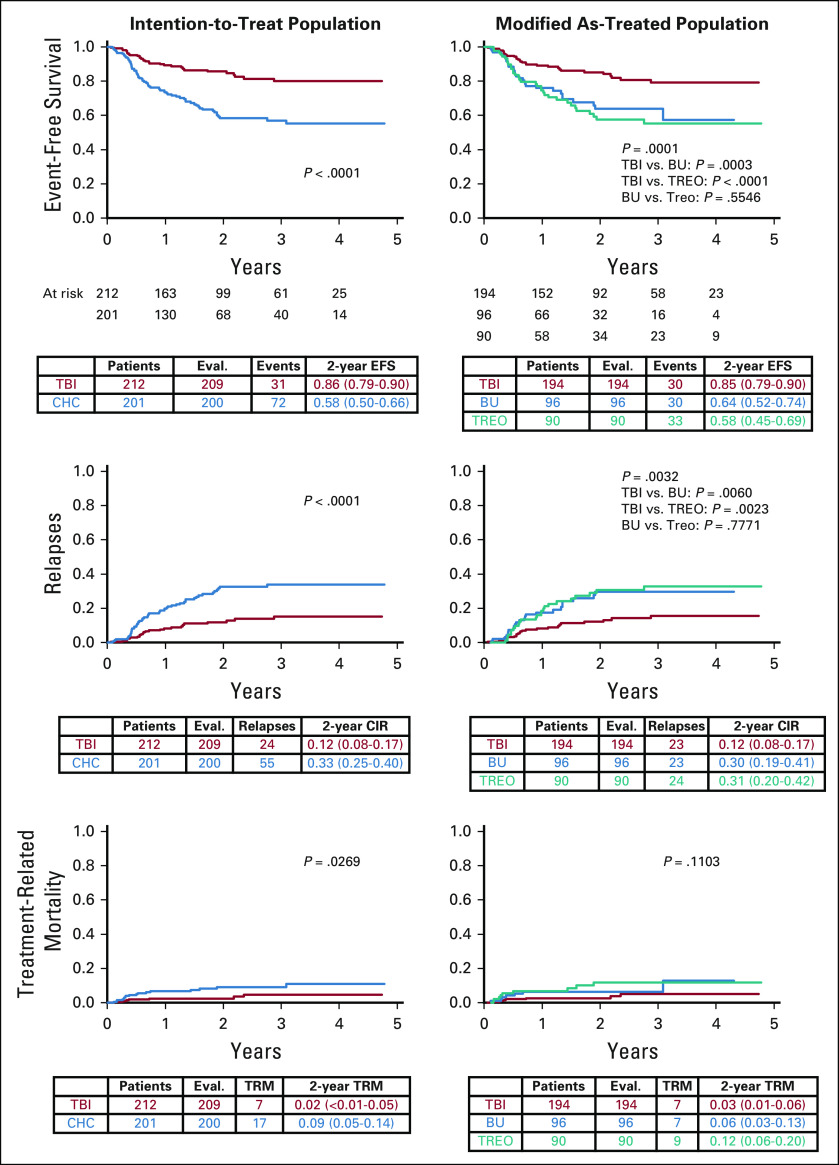
Secondary end points. BU, busulfan; CHC, chemo-conditioning; CIR, cumulative incidence of relapse; EFS, event-free survival; OS, overall survival; TBI, total body irradiation; TREO, treosulfan; TRM, treatment-related mortality.

**FIG 4.**
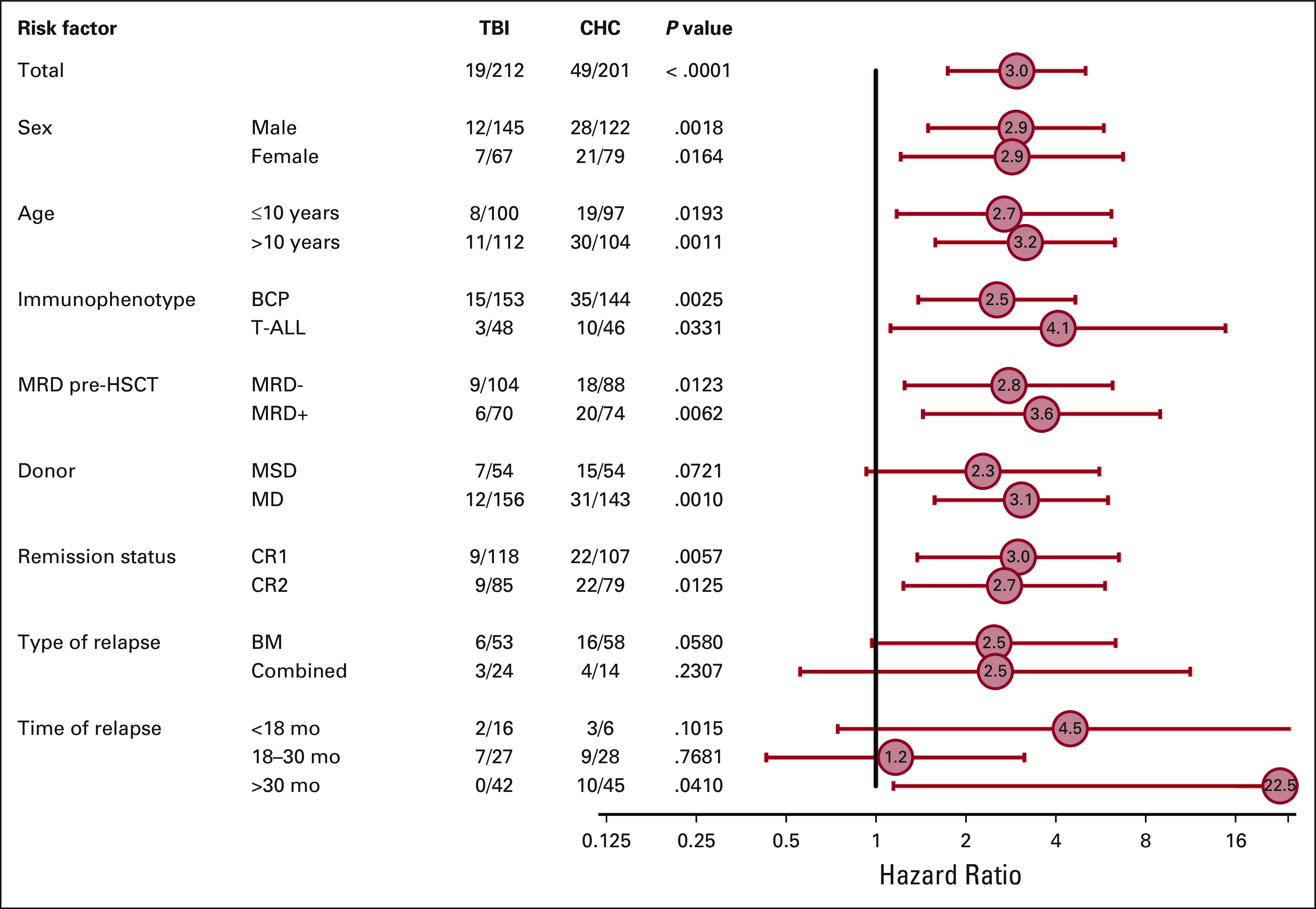
Forrest plot showing subgroup analyses of overall survival by risk factor and conditioning regimen (ITT population). ALL, acute lymphoblastic leukaemia; BM, bone marrow, CR1, first complete remission (below 5% of morphological blasts in bone marrow; no active extramedullary disease); CR2, second complete remission; CR3, third complete remission; HSCT, haematopoietic stem cell transplantation; MD, human leukocyte antigen (HLA)-compatible (nine or 10 out of 10 allelic matches) related or unrelated matched donor; MRD, minimal residual disease; MSD, HLA-identical sibling donor; TBI, total body irradiation.

In multivariable analyses, conditioning regimen reached statistical significance for OS (hazard ratio [HR], 3.1; 95% CI, 1.7 to 5.7; *P* = .0003), EFS (HR, 2.8; 95% CI, 1.7-4.6; *P* < .0001), and relapse rate (HR, 2.5; 95% CI, 1.4 to 4.4; *P* = .0001) (Table 3). EFS was lower for patients transplanted in CR2 versus CR1 (HR, 1.7; 95% CI, 1.0-2.7; *P* = .0371). Surprisingly, the presence or absence of MRD positivity pretransplant assessed by flow cytometry or PCR did not significantly influence OS or EFS in subgroup or multivariable analyses.

**TABLE 3.**
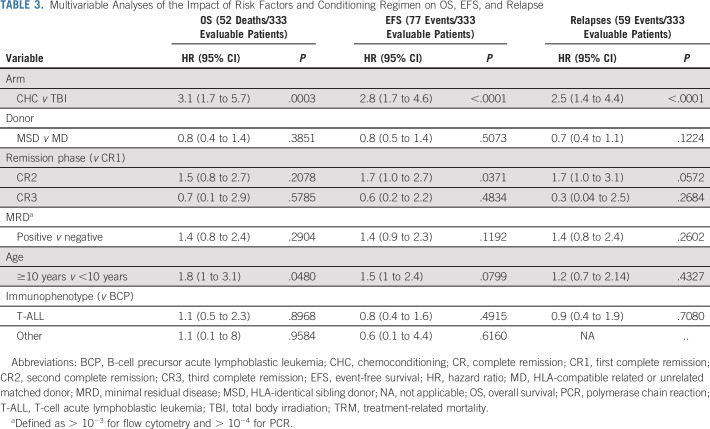
Multivariable Analyses of the Impact of Risk Factors and Conditioning Regimen on OS, EFS, and Relapse

## DISCUSSION

FORUM is the largest international, multicenter, randomized trial comparing TBI plus etoposide versus myeloablative chemoconditioning regimens (consisting of fludarabine, thiotepa, and either intravenous busulfan or treosulfan) in pediatric patients with high-risk ALL. Despite several hurdles in initiating the trial in over 20 countries and 5 continents, FORUM was made possible by collaboration between international study groups. Patients underwent allogeneic HSCT according to standardized transplant indications, donor selection criteria, stem cell source, GVHD prophylaxis, and conditioning regimen. Investigators were given recommendations for monitoring MRD and intervention strategies to reduce MRD pretreatment and post-transplant. Strong random assignment compliance, rigorous data collection, and careful, prompt interim analysis allowed detection of the inferiority of chemoconditioning to TBI plus etoposide.

In a retrospective trial, it was shown that chemoconditioning can supplant TBI at least in some young patients with ALL and favorable prognosis pre-HSCT (CR1 or age < 12 years).^15,21^ In a recent retrospective analysis of over 3,000 pediatric patients with ALL, no significant difference in OS was observed between patients in CR1 transplanted after TBI versus chemoconditioning; however, in patients transplanted in CR2, the TBI arm had significantly better OS, EFS, relapse incidence, and TRM.^22^ A similar trend was observed in our subgroup analyses; however, our study was not powered to assess statistical significance in a sample size of 413 patients. Highly encouraging 2-year OS and EFS of 0.91 and 0.91, respectively, were observed for patients transplanted in CR1 after TBI, which we believe is the highest survival rate reported in HSCT trials in childhood ALL.

Patients receiving TBI had a significantly lower risk of relapse and TRM than those given chemoconditioning. This resulted in early termination of random assignment.

The majority of post-HSCT leukemic relapses in children occur within 24 months; thus, a dramatic change in Kaplan-Meier estimates of relapse with further follow-up is unlikely. In part, the difference in relapse risk between arms could be detected early due to the low rate of TRM (higher survival rates yield a larger pool of patients to potentially relapse). There was a plateau in the rate of relapse with TBI plus etoposide after 2.5 years, whereas an extended relapse cascade was apparent with chemoconditioning. The lack of an asymptote in relapse rate after chemoconditioning suggests that between-group differences may continue to widen over time. It is unlikely that secondary malignancies after TBI (which in some studies have caused survival curves to cross)^23^ could jeopardize the survival advantage of TBI plus etoposide.

Randomized, international studies are a crucial, up-to-date evidence base supporting clinicians and patients in balancing treatment risks and benefits. We recommend TBI plus etoposide conditioning for patients > 4 years old with high-risk ALL undergoing allogeneic HSCT. However, TBI is not always an option because of lack of facilities, young age, or comorbidities. Fludarabine, thiotepa, and either busulfan or treosulfan have shown high efficacy compared with other preparative regimens in previous studies of pediatric HSCT.^16,24^ Moreover, TRM with chemoconditioning in FORUM was low versus that previously reported.^25,26^ However, relapse incidence was still high, and the best chemotherapy-based preparative regimen for patients ineligible for TBI is unknown.^22,27,28^

Despite variations including center size and heterogeneity of supportive care among the participating sites, TRM incidence was lower in FORUM than in other reports.^25,29,30^ Furthermore, established risk factors for post-transplant relapse (ie, age at transplantation, leukemic phenotype and molecular aberrations, site of relapse, MRD pretransplant, donor type, and stem cell source)^31-33^ did not significantly impact outcomes. In multivariable analyses, only remission status (ie, CR1 *v* CR2 or above) and conditioning type influenced OS and EFS. Add-on studies will explore the impact of busulfan, treosulfan, and antithymocyte globulin pharmacokinetics and pharmacogenomics on relapse rate.

We and others have previously demonstrated that high MRD levels pretransplant increased post-transplant relapse risk.^34,35^ Surprisingly, MRD level did not affect EFS in FORUM, probably because of patients' favorable MRD profile. It was a strong mission in our study to reduce MRD to below 10^−3^ with several treatment modalities including bispecific antibodies, inotuzumab, nelarabine, and other individualized methods, always bearing in mind that additional chemotherapy might increase the risk of TRM (although this was not the case). Indeed, most patients had undetectable or very low levels of MRD in pretransplant marrow aspirates. Further analyses by pretransplant MRD will be conducted.

In FORUM, relapse after transplant was associated with a low possibility of cure, which negated our premise that such patients would be salvageable by a second HSCT using TBI. This approach was associated with high TRM. The low number of patients who received chimeric antigen receptor T-cell therapy for post-transplant relapse (n = 10) does not allow conclusions to be drawn. However, emerging cell and immune therapies may offer the opportunity for rescue going forward, with potential for deep remissions and higher survival.

The higher probability of EFS observed in patients experiencing grade 2 aGVHD suggests that—at moderate severity—aGVHD is associated with a graft-versus-leukemia effect and protects from leukemia recurrence.^34-36^

Our findings cannot be generalized to centers with limited or no access to TBI. Another limitation is the relatively short median follow-up (2.1 years). Early random assignment closure limited sample size, making the prespecified primary analysis infeasible. The conditional power of the study was assessed to explore the likelihood that noninferiority could be concluded with a sample size of 1,000; which was found to be extremely low.

Recruitment without random assignment is ongoing as several questions of special interest require longer follow-up, that is, effect of conditioning on gonadal function, final height, organ functions, individual genetic variabilities, and secondary malignancies.^12,37-39^

The composite end point of 2-year GVHD-free, relapse-free survival of 72% (95% CI, 65%-78%) following TBI plus etoposide and 51% (95% CI, 43% to 58%; *P* = .0003) following chemoconditioning might be a benchmark for future investigations, accounting for second malignancy risk.

In conclusion, pediatric patients with high-risk ALL who received myeloablative TBI plus etoposide prior to HSCT had a significantly better survival and lower relapse risk and TRM versus patients who received myeloablative chemotherapy.

## DATA SHARING STATEMENT

Individual participant data (pseudonymized with an individual unique patient number) that underlie the results reported in this article, and the informed consent forms for different age groups, will be made available for individual participant data meta-analyses beginning 12 months and ending 36 months. A proposal to the corresponding author will be sent with a signed data access agreement. The study protocol and statistical analysis plan are available on the website of the principal investigator and on the study site of ClinicalTrials.gov. All other related documents are available on request to Prof. Christina Peters as international study coordinator of the FORUM trial.
